# OLSVis: an animated, interactive visual browser for bio-ontologies

**DOI:** 10.1186/1471-2105-13-116

**Published:** 2012-07-10

**Authors:** Steven Vercruysse, Aravind Venkatesan, Martin Kuiper

**Affiliations:** 1 Department of Biology, Norwegian University of Science and Technology, Trondheim, Norway

**Keywords:** Bio-ontologies, Visualisation, Browsing, Web application

## Abstract

**Background:**

More than one million terms from biomedical ontologies and controlled vocabularies are available through the Ontology Lookup Service (OLS). Although OLS provides ample possibility for querying and browsing terms, the visualization of parts of the ontology graphs is rather limited and inflexible.

**Results:**

We created the OLSVis web application, a visualiser for browsing all ontologies available in the OLS database. OLSVis shows customisable subgraphs of the OLS ontologies. Subgraphs are animated via a real-time force-based layout algorithm which is fully interactive: each time the user makes a change, *e.g.* browsing to a new term, hiding, adding, or dragging terms, the algorithm performs smooth and only essential reorganisations of the graph. This assures an optimal viewing experience, because subsequent screen layouts are not grossly altered, and users can easily navigate through the graph. URL: http://ols.wordvis.com

**Conclusions:**

The OLSVis web application provides a user-friendly tool to visualise ontologies from the OLS repository. It broadens the possibilities to investigate and select ontology subgraphs through a smooth visualisation method.

## Background

Ontologies constitute an increasingly important knowledge resource. In the biomedical domain the engineering of ontologies is predominantly organised by the Open Biomedical Ontology (OBO) Foundry [1]. Ontologies arrange terms hierarchically, connected by relationships in directed acyclic graphs. OBO ontologies represent formalised biological knowledge and are broadly used in the analysis and interpretation of experimental results, *e.g.* by linking Gene Ontology (GO) terms [2] to gene sets [3,4]. Ontologies provide also an important resource to find accurate terms for use in scientific reports.

Many tools are available for browsing ontologies (see [5,6]). Several of them are integrated in systems dedicated to analyse specific data sets (*e.g.* calculating overrepresented GO categories in a gene list: GOrilla [7], agriGO [8], and GOTermFinder [3]). Other tools are designed for more general-purpose ontology exploration, such as QuickGO [9], AmiGO [10], or NCBO’s FlexViz [11]. Some of these ontology viewers are text-based, *i.e.* they use a folder/subfolder-interface to explore hierarchies (*e.g.* AmiGO [10], MGI GO Browser [12]). However, many ontologies feature multiple-inheritance: they have terms that are linked to more than one parent. This multiple-inheritance is more clearly visualised in a two-dimensional display, with nodes and connectors in between. For instance, the Ontology Lookup Service (OLS) offers static images that clarify better how terms are positioned and related to adjacent terms in the hierarchy, and it provides this unified interface for the browsing of 79 bio-ontologies [13]. Also, the NCBO BioPortal features the graph browser FlexViz, which draws subgraphs from 293 ontologies and allows clicking on terms to bring up its local environment (*e.g.* child or parent terms) [11]. FlexViz is one of the most powerful viewers currently available. But despite the added flexibility and user-interaction support, this graphical browser may feel rigid and sometimes confusing, because it only shifts between static, pre-calculated, and often sub-optimal configurations. The addition of new terms may therefore result in large graph reorganisations that are often hard to follow.

One can easily experience why we consider the NCBO Bioportal’s FlexViz not an optimal viewer even in simple use scenarios, by trying for example the following exercise in FlexViz: open the ontology ‘Gene Ontology’, search for ‘mitochondrion’, and then expand some terms upward towards the root, *e.g.* ‘intracellular membrane-bound organelle’ and then ‘intracellular organelle’. When doing so, one is confronted with terms moving all over and far out of the viewport, with the viewport shifting over large distances. This is caused by many terms being placed next to each other on a too wide hierarchy level. Much of the overview is lost, and an attempt to regain some of it back by zooming out will leave the node labels too small to read. Using other layout algorithms than the default one (‘tree layout’) seems also less than satisfactory.

Although FlexViz constitutes an interesting first step towards a fully flexible and user-friendly browsing experience, it leaves room to explore alternative approaches to ontology visualisation. We therefore investigated if the use of a fundamentally different layout method would give a better user-experience for the general-purpose browsing of ontologies. We chose to implement a *real-time*, *force-based* layout algorithm, which can organise nodes and connections globally and dynamically. First, it uses a ‘minimum energy’ principle, ensuring that nodes and connection-structures are distributed optimally relative to each other in the available screen space. Second, it immediately responds when (and as long as) the user interacts with the graph, updating the nodes’ positions continuously until a new optimal configuration is reached.

## Results and discussion

Web-based ontology visualisers are largely used for browsing and to analyse the placement of a given term in an ontology. They help to get a grasp of the local environment of a term of interest or to view terms that form the connection to the root term (path to the root). As bio-ontologies are getting increasingly complex, browsing through them requires a visualiser that offers more intuitive functionalities such as the autosuggestion of terms, an ‘undo’ function, filtering for relationships and additional functions that facilitate smooth and user-friendly browsing. The visualisers that are currently available only have some of these characteristics, and often show limitations with respect to browsing speed, scalability issues, context-based display of a term’s environment, or overall user interaction support. This prompted us to create the web application *OLSVis*: a fast, interactive visualiser to explore OLS ontologies based on minimal and smooth relayouts. OLSVis exploits the speed and ease-of-use of the WordVis application [14,15]. Inspired by the Ontology Lookup Service, we applied the concept of a term’s local environment (child terms and path to the root [13]) as the basic viewing unit for the visualiser. We illustrate the advantages of OLSVis through three use cases, exemplifying both the added functionalities and the enhanced user-experience that OLSVis brings to ontology visualisation. Use Case I demonstrates a general overview of the features of OLSVis, highlighting its interactive environment using the Gene Ontology. Use Case II illustrates an approach to view common ancestor terms shared between two Gene Ontology terms; and Use Case III demonstrates the visualisation of the local neighbourhood of a protein.

### Use case I: Browsing ontologies in OLSVis

**Figure 1:**
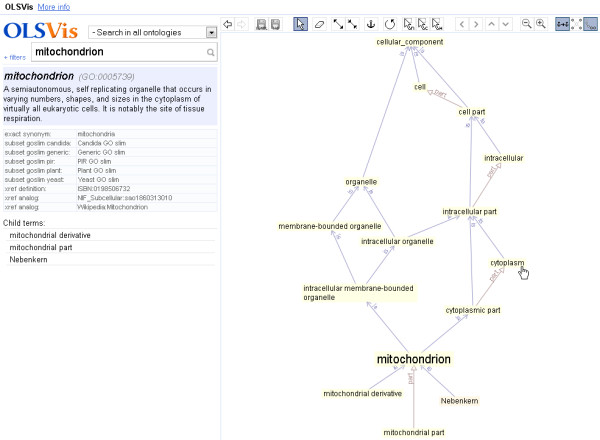
**Screenshot of the OLSVis web application.** OLSVis has been used to search the Gene Ontology term ‘mitochondrion’ (see search box). The graph panel on the right shows the term’s child, parent and ancestor terms linked by various relationship types. Clicking any term launches a new search focused on that term. The left panel shows ontology details for the displayed term.

The first use case illustrates how OLSVis can make ontology browsing more intuitive: A user is interested in the placement of the term ‘mitochondrion’ in the Gene Ontology hierarchy. She can proceed in two ways: a) select the ontology of choice and then search for the chosen term, or b) do a direct search for the GO term ‘mitochondrion’. Autosuggestion enables her to perform a quick selection of the term from the autosuggest list. Autosuggestion also highlights the occurrence of the chosen term in other ontologies. The user selects the GO entry, in this case ‘mitochondrion’ (GO:0005739) and OLSVis shows the official GO term centered in the visualiser, along with its child terms and all paths of ancestor terms up to a GO root term (Figure 1). The display of the local environment of the term is dynamic and the visualiser allows the use of various features to further refine the display (see the toolbar). For instance, the ‘Eraser’ tool can be used to hide unnecessary terms from the display panel. In some cases the relation names are abbreviated for a clearer view and displayed in full by mouse-hovering. Parts of the graph can be made less/more compact by increasing/decreasing the length of connectors. Also, similar to modern map-applications, OLSVis supports moving the graph by dragging its background and zooming by mouse scrolling. Furthermore, a ‘filters’ panel is provided to assist the user in narrowing or broadening the search space. In Figure 1 both ‘is_a’ and ‘part_of’ relations are shown.

Other improvements that OLSVis provides concern the animation and presentation of terms after specific user actions. For instance, clicking on ‘cytoplasm’ will shift the display into cytoplasm’s local environment (the children and all ancestors of the term ‘cytoplasm’). The algorithm switches between local environments by gently pushing out terms and inserting new terms, which allows a user to easily keep track of the changing display. A button on the toolbar may be used to prevent automatic removing of nodes. Its dynamic layout algorithm and the additional graph interaction tools all contribute to the user-friendliness of OLSVis. Furthermore, OLSVis allows the user to save the local environment in XGMML format that may be imported in network building tools such as Cytoscape [16,17]. Alternately, the user can obtain the list of nodes and relationships in the current view in a tab-delimited file.

### Use case II: Identifying shared ancestor terms between two ontology terms

**Figure 2:**
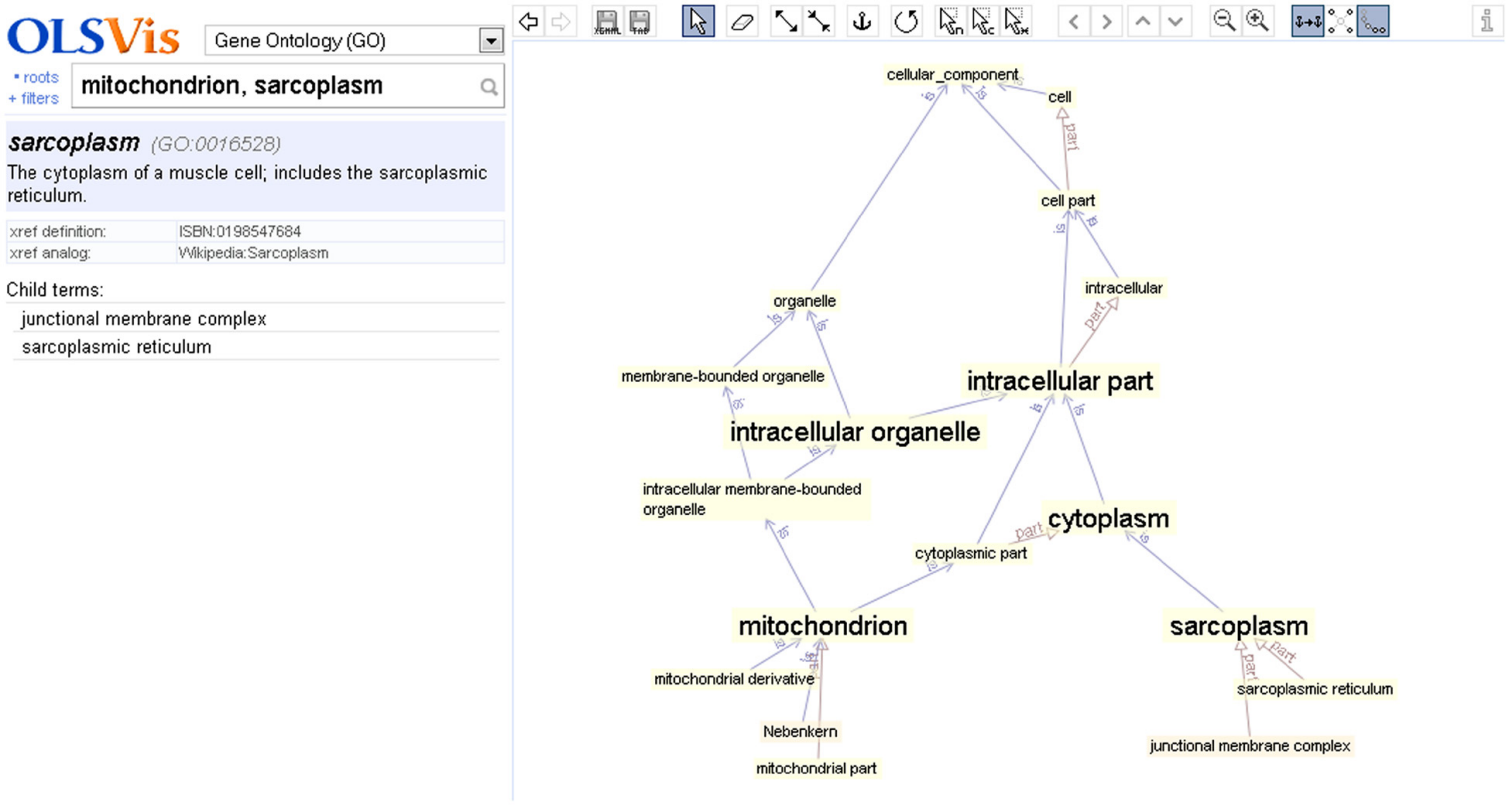
**OLSVis screenshot of use case II.** The canvas shows the combined local environments of two search terms, their paths to the root and thereby the relatedness between the terms. The search box in the left panel shows the two terms. Also a number of terms were ‘anchored’ by the user.

Suppose a user wants to identify the common ancestry between two different terms, in order to assess their relatedness. Use case II shows an example based on the cellular components ‘mitochondria’ and ‘sarcoplasm’. Here the user first selects ‘Gene Ontology’ from the ontology list and then enters two terms in the search box, separated by a comma. OLSVis reads both text strings as separate terms, matches them to their respective terms in the selected ontology, GO, and then displays a merged view of their local environments. Figure 2 shows the terms that hereby are displayed, linking ‘mitochondria’ and ‘sarcoplasm’ and showing their shared connections. Additionally, for customised visualisation, shared terms could be repositioned and fixed by using the ‘Anchor node’ functionality. Non-anchored terms will slide to new optimal positions. This example demonstrates the potential of OLSVis in displaying environments for multiple terms which is currently not available in any other visualiser.

### Use case III: Visualising the local neighbourhood of a protein

**Figure 3:**
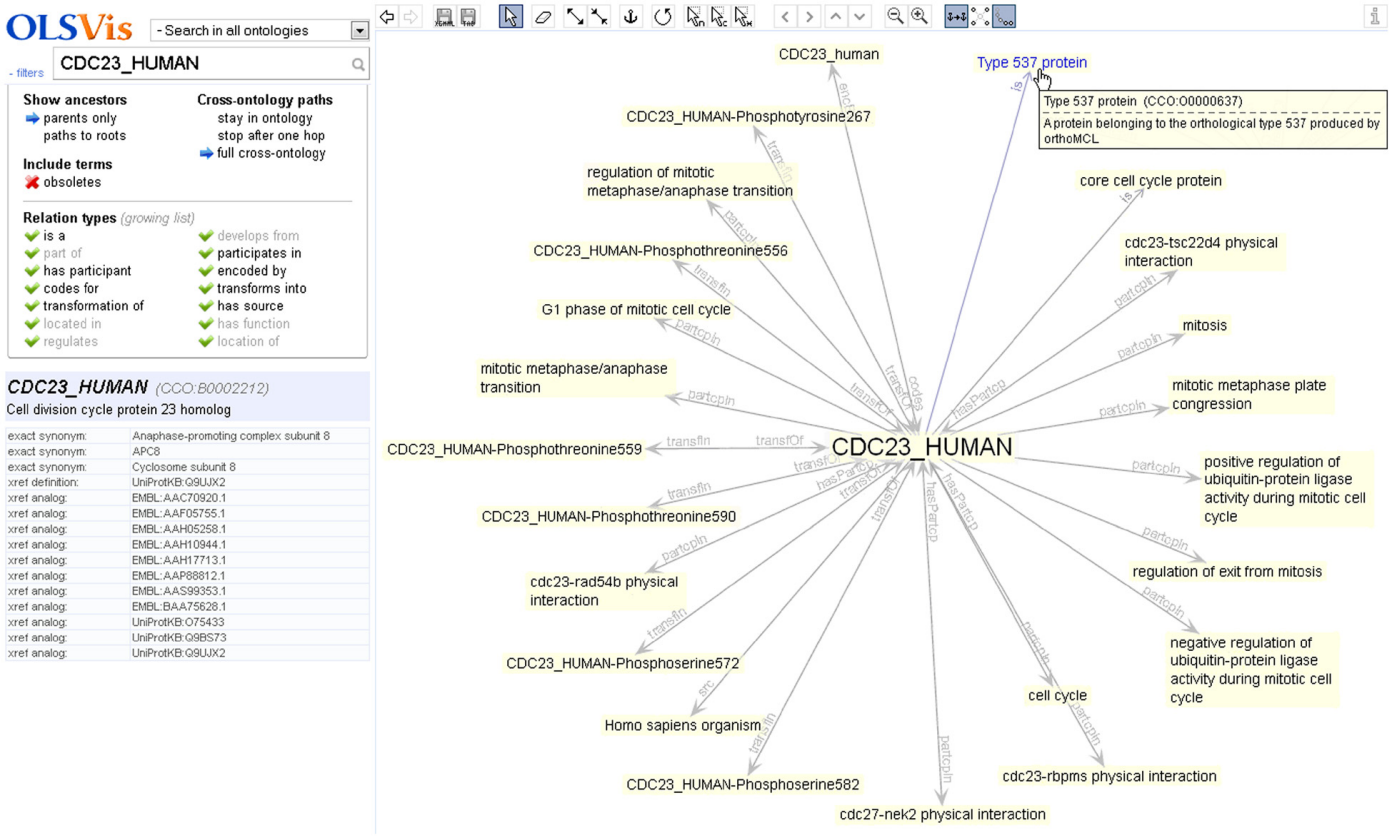
**Screenshot illustrating use case III.** The canvas shows the local neighbourhood of a CCO term. The left panel shows the term name in the search box and the pop-out panel with filter settings. The filter setting enables the display of only parent terms.

Biologists are often interested in understanding the various attributes of a particular protein such as protein modifications, biological functions, or protein interactions. Use case III illustrates how OLSVis can be used for visualising the local neighbourhood of a protein. In this example the protein is cdc23 (*H. sapiens*). The user enters the string ‘cdc23’ and the autosuggestion list shows a number of matches from the Cell Cycle Ontology (CCO) [18]. Selection of the term ‘cdc23_HUMAN (CCO:B0002212)’ displays the local neighbourhood of this term whilst providing a warning message that alerts the user as to the large number of terms associated with the chosen protein. When browsing large ontologies (*e.g.* CCO), a user usually has to deal with performance issues as the visualiser may actually fail to load the subgraph due to its size. Instead, OLSVis loads up to 500 terms smoothly and if more it gives a notification to the user. The user is suggested to use the filter panel to narrow the search space for improved performance and viewing. For example, clicking on ‘parents only’ will update the current view with a simplified graph (Figure 3). Alternatively, a number of relation types could be filtered away. Here we note that CCO includes bidirectional relationships, so leaving some out can clarify the intended parent–child hierarchy. The user may then choose to save the current display in formats provided by OLSVis. For instance, biologists to a large extent still work on spreadsheets where they periodically associate a particular protein of interest with an ontological term. In such cases, saving the current view in a tab-delimited format makes it easier for them to use the terms associated with a protein in their annotation work.

## Implementation

For the client-side of the software, we used the modern web technologies of JavaScript and the new HTML5 standard. In contrast to traditional Flash-objects or Java-applets, which are isolated objects in the web page, JavaScript and HTML5 make it possible to create animations that are fully connectable with other elements on the web page, and that require no extra browser plugins. HTML5 defines the < canvas > HTML-element, basically a rectangular empty space on the web page, onto which JavaScript code (which is included in the web page) draws basic shapes like lines, circles, text, etc. Note that the older SVG (Scalable Vector Graphics) technology requires computationally expensive (slow) DOM-updates; therefore only canvas is appropriate for smooth animation of large graphs. Because a sufficiently powerful JavaScript library for animated graph browsing did not exist yet, we wrote one from scratch: GraphVis. We first applied GraphVis in the webapp WordVis [14,15], which visualises WordNet, a lexical database of English [19].

### Layout engine

We applied our GraphVis layout module to the exploration of ontologies in OLSVis, and upgraded it among others with hierarchical layout for parent/ancestor terms, see Figure 1. When the user searches for an ontology term, OLSVis will by default show it together with its child terms and parent terms up to the ontology root(s), see Figure 1. After initial placement of ontology terms, OLSVis uses a real-time force-based layout algorithm that gently moves the terms towards more optimal positions. The algorithm is explained in [14] and [15]. It models nodes as repelling, electrically charged rectangles. This distributes them over the screen, prevents them from occupying the same space (if possible), and prevents term labels from overlapping. Connections are modelled as mechanical springs, which hold nodes together and which may be given a specific preferred-length in order to create a certain global structure in the graph. Connections may also have a preferred orientation (*e.g.* down-to-up for ‘is a’ links). This layout is fully interactive: each time the user makes a change (such as focusing on a new term, hiding, adding or dragging terms, changing connection lengths), it smoothly yet minimally reorganises the graph. This assures an optimal viewing experience that minimises each operation’s effect on graph reorganisation, and maximises the user’s ability to keep track of changes and comprehend the new lay-out.

### Data source

OLSVis visualises the contents of the OLS database [13,20,21], which holds around 80 bio-ontologies and over 1 million concepts. OLSVis uses a local copy of OLS‘ publicly available database, in order to provide a smooth visualisation with fast response times. Only via a local copy placed on OLSVis’ server can the node environments be calculated sufficiently fast. The use of the OLS web-service to retrieve data proved to be painfully slow, because each mouse click required several web-service queries, which typically resulted in total query times of several tens of seconds. EBI updates the OLS database weekly by polling its ontology providers through the CVS and SVN version control systems. OLSVis detects OLS’ updates automatically and then updates its local copy. In addition, a number of table-indexes and pre-calculated fields are added to enable the speed of OLSVis. On the server-side of OLSVis, PHP scripts translate client-side requests into custom queries on the local MySQL database. Note that the web-application’s front-end is designed independent from the database back-end. Given software that would be able to calculate node-environments (filterable paths-to-roots) in reasonably short times, the visualiser would be usable also for other semantic resources.

### Term searching

While the user types one (or several) terms or identifiers (*e.g.* ‘mito’ or ‘PO:0009001’) in the search box, a selection of best known matches is shown in a pop-out list. This includes preferred terms as well as their synonyms. For each autosuggested term, the ontology’s (short)name and identifier is shown, and mouse-hovering shows its ontology’s full name. Autosuggestions can be confined to a single specific ontology by selecting one from the drop-down list. Pressing ‘Enter’ in the search box will display the term that is selected in the autosuggestion list. If the user has *no term selected*, OLSVis will take the *first* term (also if the autosuggestion list has not appeared yet).

### Basic visualisation

The chosen term is then *expanded*: it is placed in the centre of the graph panel, amidst its *local environment* of child terms and parent terms, and connected with further ancestors up to the ontology root(s). This configuration is inspired by OLS’ static images [13]. Child terms are ordered in a half circle under the expanded term; ancestors are put in hierarchical levels above it. Relations are shown as labelled arrows; their lengths are adjusted for good hierarchical positioning. After initial placement, the visualiser slides terms to more optimal positions via real-time animation; hereby the graph ‘feels’ and behaves as if terms are repelling electric charges (or repelling magnets) that are connected over mechanic/elastic springs. This creates a layout that minimises term overlap. In addition, the connecting arrows undergo a small north–south orienting force to enhance a hierarchical alignment of terms. The visible graph is fully customisable: see the toolbar in Figure 1 or the online description for mouse/keyboard shortcuts. It has undo/redo history, and terms can be dragged and pushed around. Clicking on any displayed term will re-centre on that term and expand its local neighbourhood. Hereby the graph is subtly reorganised via real-time animation, and is transformed into the new term’s local environment (by addition and removal of terms). This enables easy and intuitive browsing through ontologies. The automatic removal of already visible terms can be turned off via the rightmost button on the toolbar. Hovering over any term makes its definition pop up. For a relation arrow, its non-abbreviated name pops up. Leaf terms (=without child terms) get a slightly orange background. The three most common relations (is_a, part_of, develops_from) get a coloured arrow. In the left panel, data for the *last* expanded term is shown: its identifier (hovering shows ontology’s full name) and definition; its synonyms, annotations and cross-references (as in the OLS database); and its child terms (each clickable to expand), to make them easier visible when there are many. When zooming in, OLSVis increases distances faster than font sizes; this is more useful and is an extra method (next to electrostatic repulsion) to counteract overlapping terms.

### Customised visualisation

A click on ‘filters’ (left of the search box) brings up a panel to set filters that prune the expanded node’s environment. For instance, any relation type can be excluded; this means that they are omitted when building *e.g.* the path-to-root. Initially the three most common relation types are listed in the panel; this list grows each time the visualiser encounters new types. Relation types that are currently in the visualiser are highlighted. The filter that hides obsolete terms also hides them in autosuggestion lists. Earlier expanded terms and their environment are by default automatically removed when clicking on a new term, but can be kept in the visualiser by turning off the rightmost toolbar button. Several toolbar tools enable further customisation of the graph. Connections can be made longer or shorter (also via Alt + scrolling up/down). Terms can be anchored to a fixed position, and anything can be removed manually via the Eraser tool.

### More features

A ‘roots’ link appears next to the search box after selecting a specific ontology. Clicking it shows and expands this ontology’s root term(s) (if defined in the OLS data), *i.e.* showing them and their child terms. This enables easy top-down ontology exploration. Multiple terms and identifiers can be searched, separated by commas. Therefore in-term commas must be preceded by a backslash, and genuine backslashes doubled. First hits from autosuggestion are then expanded. When a term’s local environment contains too many terms (this happens with application ontologies such as the Cell Cycle Ontology [18]), OLSVis will only show the first 500 terms and will suggest using filters. OLSVis supports URL-shortcuts:

(1) A term or identifier can be expanded directly via URLs like: *ols.wordvis.com/q = GO:0005739*, or *…/q = mito*. The part after */q =* will be put in the search box and the first term that would have been autosuggested will be expanded.

(2) A specific ontology can be preselected via a URL like: *ols.wordvis.com/ont = GO*. The part after */ont =* is the ontology’s short name from the selection list. This is a shortcut for users that are mainly interested in a specific ontology.

(3) ‘q’ and ‘ont’ can be combined like: *…/ont = GO&q = mitochondrion,sarcoplasm* , which also illustrates a multi-term query.

(4) Some ontologies use non-standard prefixes in term-identifiers (GO has ‘GO:’, but ZFA may use ‘ZFS:’, and NEWT has none), so identifiers may be disambiguated by adding their ontology’s short name as prefix, e.g. *…/q = NEWT:1234*, or *…/q = ZFA:ZFS:0000019*.

Terms in the graph can be right-clicked for more options. The visible graph can be exported to an XGMML file (eXtensible Graph Markup and Modeling Language) and can subsequently be imported in Cytoscape [16,17] for further analysis. There, node labels will show the term names, and ‘ontID’ attributes store the ontology identifiers. In addition, nodes and relations can be exported to a tab-delimited text file.

### Comparison with other visualisation tools

The utility and performance of OLSVis was assessed in comparison to other tools commonly used for ontology visualisation, including some biological data analysis tools that have visualisation components integrated in them, as listed in Table 1. The evaluation addressed a number of criteria, including tool functionality (*e.g.* support of multiple term searching); scalability (*e.g.* handling of large numbers of terms); and some aspects that capture user-friendliness and intuitiveness of browsing (*e.g.* context-dependent browsing). The table shows that some of OLSVis’ features are not provided by any other visualiser, and that the other tools only support a subset of what OLSVis offers. Clearly, OLSVis offers the most interactive visualisation environment. FlexViz ranks well too, as it also provides a relatively high level of user-interaction; however, OLSVis makes more efficient and intuitive use of the available screen space.

**Table 1:** Comparison of OLSVis and other two-dimensional ontology visualisers

	**OLSVis**	**OLS**	**FlexViz**	**OntoViz**	**IsaViz**	**GOSurfer**	**GOMiner**	**OntoTrack**	**OBO-Edit**	**QuickGO**	**AmiGO**
**Full Dynamic layout method**	✓		✓(semi) ^1^	✓(semi) ^1^				✓(semi) ^1^	✓(semi) ^1^		
**Layout user-interaction**	Interactive; + continuous optimisation^2^		one-by-one dragging	one-by-one dragging	highlighting a selected branch			one-by-one dragging	one-by-one dragging		
**Hierarchy layout**	ancestors: layered; children: circular	ancestors: layered; OR: parents + children	several^3^	ancestors + children: layered	graph-view or radar-view	tree view	ancestors + children: layered	ancestors + children: layered	ancestors + children: layered	ancestors	subfolders
**Term searching**	✓	✓	✓	✓	✓		✓	✓		✓	✓
**Multiple term search**	✓										
**Auto-suggest**	✓	✓	✓							✓	
**Filters**	✓		✓					✓			
**Undo**	✓	✓^4^	✓	✓	✓			✓		✓^4^	✓^4^
**Context dependent display**^5^	✓										
**Click on term expands it**	✓	page reload	✓	✓				✓	✓		✓
**Path to root**	✓	✓	✓	✓	✓	✓	✓	✓	✓		✓
**Simple tree view**		✓	✓	✓		✓	✓	✓	✓		✓
**Handling 500 terms**	✓						✓				
**Term source**	OLS (79 ontologies)	OBO (79 ontologies)	NCBO (293 ontologies)	OWL ontologies	RDF graph visualiser	GO (1)	GO (1)	OWL ontologies	OBO ontologies	GO (1)	GO (1)
**Web tool (Technology)**	✓ (Javascript)	✓ (images)	✓ (Flash)	–(Protégé)	–(Java)	–(.exe)	–(Java)	–(Java)	–(Java)	✓ (HTML)	✓ (HTML)

^1^: Only shifting between static configurations. ^2^: Also during the several editing functions (which are displayed on OLSVis’ toolbar). ^3^: Each with their own shortcomings, see ‘Background’. ^4^: Browser’s back button. ^5^: The visualiser can optionally clean up nodes that are not in the latest expanded node’s “environment”.

Comparison with: OLS [13], FlexViz [11], OntoViz [22], IsaViz [23], GOSurfer [24], GOMiner [25], OntoTrack [26], OBO-Edit [27], QuickGO [9], AmiGO [10]).

## Conclusions

OLSVis was created to improve the exploration of bio-ontologies. Other visualisers like FlexViz, may feel rigid and sometimes confusing, because the addition of new terms may result in largely rearranged term displays. OLSVis demonstrates that the user experience for ontology exploration can be substantially improved by using real-time animation of force-based graph relayout, and by providing improved user interaction on the graph’s structure. This new webapp provides the scientific community with a versatile and more user-friendly tool to explore ontologies and to find related and more precise ontology terms.

## Availability and requirements

· **Project name:** OLSVis

· **Project home page:**http://ols.wordvis.com

· **Operating system:** Platform independent

· **Programming language:** JavaScript, PHP, (MySQL)

· **Other requirements:** Modern browser: recent version of Firefox, Chrome, Opera, Safari or Internet Explorer. (IE 8 not recommended; please upgrade to IE 9, which supports ‘canvas’ and thus is much faster). No browser plugin needed.

· **License:** The web-application is freely accessible for use.

· **Any restrictions to use by non-academics:** No specific restrictions.

## Abbreviations

CCO: Cell Cycle Ontology; CVS: Concurrent Versions System; EBI: European Bioinformatics Institute; GO: Gene Ontology; NCBO: National Center for Biomedical Ontology; OBO: Open Biomedical Ontology; OLS: Ontology Lookup Service; SVN: Apache Subversion; XGMML: eXtensible Graph Markup and Modeling Language.

## Competing interests

The authors declare that they have no competing interests.

## Authors’ contributions

SV designed and programmed the software and drafted the manuscript. AV helped testing the software and draft the manuscript. MK helped conceive the web application and draft the manuscript. All authors read and approved the final manuscript.
